# Evaluation of volumetric modulated arc therapy (VMAT) with Oncentra MasterPlan^® ^for the treatment of head and neck cancer

**DOI:** 10.1186/1748-717X-5-110

**Published:** 2010-11-22

**Authors:** Judith Alvarez-Moret, Fabian Pohl, Oliver Koelbl, Barbara Dobler

**Affiliations:** 1 Department of Radiotherapy, Regensburg University Medical Center, Regensburg, Germany

## Abstract

**Background:**

Several comparison studies have shown the capability of VMAT to achieve similar or better plan quality as IMRT, while reducing the treatment time. The experience of VMAT in a multi vendor environment is limited. We compared the plan quality and performance of VMAT to IMRT and we investigate the effects of varying various user-selectable parameters.

**Methods:**

IMRT, single arc VMAT and dual arc VMAT were compared for four different head-and-neck tumors. For VMAT, the effect of varying gantry angle spacing and treatment time on the plan quality was investigated. A comparison of monitor units and treatment time was performed.

**Results:**

IMRT and dual arc VMAT achieved a similar plan quality, while single arc could not provide an acceptable plan quality. Increasing the number of control points does not improve the plan quality. Dual arc VMAT delivery time is about 30% of IMRT delivery time.

**Conclusions:**

Dual arc VMAT is a fast and accurate technique for the treatment of head and neck cancer. It applies similar number of MUs as IMRT, but the treatment time is strongly reduced, maintaining similar or better dose conformity to the PTV and OAR sparing.

## Background

Intensity modulated radiotherapy (IMRT) is the standard external radiotherapy technique to treat head-and-neck tumors because of the clinical benefits of parotid glands and spinal cord sparing [1-5]. Volumetric arc therapy (VMAT) is an extension of IMRT, which allows irradiation with simultaneously changing multileaf-collimator (MLC) position, gantry position, and dose rate [6]. Various treatment planning studies have been published showing the potential of VMAT to reduce treatment time without compromising plan quality compared to IMRT [7-14].

The result of VMAT optimization may, however, depend on the choice of various plan parameters, e.g. the number of arcs, the maximal delivery time or the gantry angle spacing between subsequent control points. Some studies showed that single arc VMAT can achieve dose distributions comparable to IMRT for prostate cancer [15-17], but for more complicated planning target volume (PTV) as it is the case in the treatment of head and neck cancer reports are contradictory. Most publications state that two or more arcs are required [15,18-20], whereas Bertelsen et al. [21] found that a single arc is sufficient to achieve plan quality comparable to IMRT.

The purpose of this study was a treatment planning comparison of IMRT and VMAT for head and neck carcinomas with different target geometries with Oncentra Masterplan^®^. The focus was to investigate the influence of various user-selectable parameters like number of arcs, gantry angle spacing and the allowed maximal delivery time on the plan quality and to identify the best parameter set for optimal combination of plan quality and treatment time.

## Methods

### Treatment planning system and equipment

A SynergyS^® ^linear accelerator (Elekta Ltd, Crawley, United Kingdom) with 6MV photons is used for IMRT and VMAT delivery. The MLC consists of 40 leaf pairs of 4 mm width at isocenter. The following VMAT specific parameters were determined: minimum and maximum number of monitor units (MU) per degree of gantry rotation (0.10 MU/° and 20.0 MU/°), minimum MU per cm leaf travel (0.30 MU/cm), maximum gantry speed (6.00 °/s), maximum leaf speed (2.4 cm/s), static minimum leaf gap (0.0 cm), dynamic minimum leaf gap (0.14 cm) and maximum nominal dose rate (500 MU/min). For the SynergyS^® ^a continuous variation of the dose rate is not allowed. Seven fixed dose rate values are available, each value is half the dose rate of the next higher value. The linac selects automatically the best combination of dose rate, gantry speed and leaf speed. Therefore, the treatment time selected in the optimization differs mostly from the delivery time. Mosaiq^® ^v1.6 (IMPAC Medical Systems, Sunnyvale, CA) is used as record and verify system.

The treatment planning for IMRT and VMAT was performed with Oncentra MasterPlan^® ^v3.3 on a 64 bit Window system with 8 GB RAM and 8-core processor. This version supports VMAT planning for Elekta treatment units with single arc, dual arc or multiple individual arcs. Energy and collimator angle are defined by the user in the Beam Modeling module. Both coplanar and non-coplanar arcs are supported, with arbitrary collimator angles. The patient anatomy and targets are defined; beams are defined specifying the isocenter, beam energy, collimator angle, and couch angle for each beam. For single arc VMAT, one beam per arc needs to be defined. For dual arc VMAT an additional beam is required and both beams must have the same beam setup, while multiple single arc plans can have a different beam setup.

The IMRT plans were optimized with the option Direct Step and Shoot (DSS). This option consists on a fluence optimization with subsequent leaf sequencing for a few iterations (the sequencer creates a number of segments equal or below the number predefined by the user). The result of this first optimization is an initial guess of the segments. Then, the leaf positions and weights are optimized with a gradient algorithm. The result is a set of MLC segments ready for delivery [22-24]. For the VMAT plans the option VMAT was used. Gantry start angle, arc length, gantry angle spacing between control points and maximum delivery time are defined by the user. The collimator angle is kept constant for each arc. When using more than one arc, the dual arc option can be used, this option groups the segments such, that the leaf movement is reduced; for example, the first arc contains the segments positioned to the right and the second arc to the left. After these parameters have been defined, a few iterations are performed to create coarse segments around the arc. A fluence optimization is performed for these segments and afterwards the fluence maps are converted in MLC segments. Cloned segments are added until the final gantry angle spacing is reached. At this point, the final segments are optimized to fulfill the DVOs and machine constraints. The continuous dose delivery is thus discretized in control points (which can be defined changing the gantry angle spacing) for dose calculation. This implies that the dose is in fact approximated as being delivered in discrete segments [25].

Oncentra MasterPlan^® ^uses a fast pencil beam dose calculation algorithm based on simplified value decomposition during optimization and allows the user to choose between pencil beam and collapsed cone for the final dose calculation. In order to improve results it is recommended to run the VMAT optimization twice. In the cases I, II and III pencil beam was used for the intermediate (i.e. first accurate) dose calculation. The same algorithm was used for the final dose calculation in these cases. For the case IV collapsed cone was used for both calculations.

Both dose volume constraints (DVC) and dose volume objectives (DVO) are available for DSS optimization, while for the VMAT planning only DVOs are allowed. A weight factor, which defines the priority of each region of interest, must be assigned to each DVO. The weights of the DVOs have an impact on the optimization process; therefore the same DVOs and weights were used for both VMAT and IMRT optimizations.

### Treatment planning study

#### Patient anatomy, planning objectives and beam set-up

A selection of patients that underwent postoperative irradiation with IMRT to treat head and neck malignancies has been included in the study. Since the aim of the study was to investigate the feasibility of VMAT with the combination of Oncentra MasterPlan^® ^and an Elekta SynergyS^® ^linear accelerator for head and neck cancer in general, rather than a plan comparison with statistical significance for only one type of cancer, four patients with different typical target geometries were chosen:

I. Patient with a carcinoma of the oral cavity, PTV 800 ccm, TNM-classification: pT4pN0. PTV encompasses submental, submandibular, and subdigastric nodes (Level I/II), the upper boarder includes the hard palate with a 1-cm margin above (no "bite block" to depress the tongue downwards was used due to missing compliance), the inferior boarder was at the level of the vocal chords. The dose prescription was 60 Gy in 2Gy fractions to the PTV.

II. Patient with a carcinoma of the hypopharynx, PTV 644 ccm, TNM-classification: pT2pN2a. The PTV encompasses level I-VI nodal stations, the upper boarder is located in the nasopharynx, and the inferior boarder includes the upper cervical esophagus because of the propensity of these cancers to spread submucosally. Dose prescription for the PTV was 55.8 Gy to be delivered in 1.8 Gy fractions.

III. Patient with a carcinoma of the oral cavity, PTV 592 ccm, TNM-classification: pT4apN1. The PTV encompasses submental, submandibular, and subdigastric nodes (level I/II) and also level III -V nodal stations because of nodal involvement, the upper boarder includes the hard palate with a 1 cm margin above (no "bite block" to depress the tongue downwards was used due to missing compliance), the inferior boarder was the costoclavicular ligament with a splitting of the target volume of the cervical nodes from the level of the vocal chords. Dose prescription was 54 Gy in 1.8 Gy fractions to the PTV.

IV. Patient with a carcinoma of the nasal septum (nasal cavity), 218 ccm, TNM-classification: pT2cN0. The PTV encompasses the retroparyngeal lymph nodes and the entire nasal cavity and ethmoid-sphenoid complex. The superior margin encompasses the cribriform plate, the inferior border includes the hard palate with a 1 cm margin. Dose prescription was 54 Gy delivered in 1.8 Gy fractions to the PTV.

**Figure 1:**
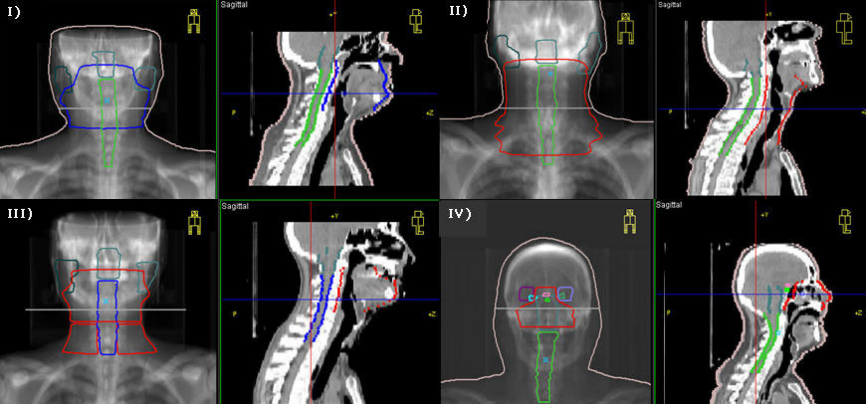
**Patient anatomy**. Patient anatomy contoured in Oncentra MasterPlan^®^.

Figure 1 shows the patient anatomy for the four selected cases.

For VMAT optimization it is not allowed to use DVCs, so both techniques DSS-IMRT and VMAT were optimized only with DVOs. For each patient, identical DVOs were applied to the organs at risk (OAR) and PTV for VMAT and IMRT. Although for patients II and III the dose prescription included an additional boost, to simplify the comparison, the study focuses only on the dose prescription to the PTV without boost. The priority was to achieve at least 95% of the prescribed dose to at least 95% of the PTV volume and to keep the dose achieved to 5% of the PTV under 107% of the prescribed dose. The tolerance dose values for each OAR can be found in table 1. The DVOs were not identical to the tolerance dose values, because it was tried to keep the dose achieved to the OARs as low as possible below these values. The whole tissue without the PTV was delineated and used as a help structure to avoid hot spots.

**Table 1:** Treatment plan comparison for patient I, II, III and IV.

	Patient I								
		**IMRT**	**s4°150s**	**d4°300s**	**d2°300s**	**d6°300s**	**d4°200s**	**d4°400s**	**Goal**

	**D_95%_**	58.2	56.6	57.9	57.7	57.4	58.0	57.6	57.0
**PTV**	**D_5%_**	61.4	62.3	61.8	62.1	62.1	61.6	62.5	64.2
	**H**	5.3	9.5	6.5	7.3	7.8	6.0	8.2	lower
	**CI (%)**	0.98	0.94	0.98	0.97	0.96	0.98	0.98	1.0
**Parotid le**	**D_50%_**	23.6	21.7	20.4	20.0	20.1	19.7	17.0	28
**Parotid ri**	**D_50%_**	22.5	20.4	19.0	20.1	19.2	18.9	17.1	28
**Brain stem**	**D_1ccm_**	43.2	44.5	44.1	42.6	42.9	44.4	44.3	50
**Spinal cord**	**D_1ccm_**	46.4	50.9	44.1	43.7	45.0	44.1	44.0	48
	**MU**	591.7	458.6	711.5	725.8	632.2	678.4	711.8	lower
	**treat. time (s)**	686	116	200	222	225	235	207	lower

	**Patient II**								

	**D_95%_**	54.2	53.2	53.9	53.7	54.0	52.9	53.9	53.5
**PTV**	**D_5%_**	57.9	58.4	58.1	58.9	58.0	58.2	59.3	60.2
	**H**	6.8	9.3	7.5	9.4	7.2	9.4	9.6	lower
	**CI (%)**	0.97	0.94	0.97	0.95	0.97	0.92	0.97	1.0
**Parotid le**	**D_50%_**	12.4	10.8	12.1	15.1	12.2	13.7	12.4	23
**Parotid ri**	**D_50%_**	10.7	17.3	11.3	16.1	11.5	13.7	11.4	23
**Brain stem**	**D_1ccm_**	33.6	30.9	29.7	31.9	30.8	37.9	30.1	41
**Spinal cord**	**D_1ccm_**	35.7	37.5	35.8	33.8	35.5	36.0	35.6	40
	**MU**	648.9	581.2	600.8	608.9	565.6	638.2	607.6	lower
	**treat. time (s)**	729	120	225	264	227	279	248	lower

	**Patient III**								

	**D_95%_**	51.5	51.2	51.6	50.5	51.3	52.0	51.4	51.3
**PTV**	**D_5%_**	55.5	55.9	55.4	56.0	55.7	55.3	55.3	57.8
	**H**	7.4	8.7	6.9	10.2	8.1	6.2	7.2	lower
	**CI (%)**	0.96	0.95	0.96	0.92	0.95	0.97	0.95	1.0
**Parotid le**	**D_50%_**	28.0	27.7	27.2	26.1	26.2	27.2	26.4	24
**Parotid ri**	**D_50%_**	27.0	24.7	24.1	24.0	24.5	23.5	24.5	24
**Brain stem**	**D_1ccm_**	36.5	35.6	31.9	38.2	31.9	31.5	31.6	43
**Spinal cord**	**D_1ccm_**	40.7	40.8	39.3	42.0	39.1	38.6	39.2	41
	**MU**	596.1	515.6	584.1	664.2	556.4	589.2	618.8	lower
	**treat. time (s)**	753	115	225	303	213	240	246	lower

	**Patient IV**								

	**D_95%_**	51.5	49.7	49.8	46.4	51.1	51.0	50.8	51.3
**PTV**	**D_5%_**	55.4	56.5	55.9	59.8	58.0	55.6	56.1	57.8
	**H**	7.2	12.6	11.3	24.8	12.8	8.5	9.8	lower
	**CI (%)**	0.95	0.90	0.92	0.74	0.94	0.94	0.93	1.0
**Spinal cord**	**D_1ccm_**	8.8	16.2	13.6	15.4	16.3	14.5	13.1	48
**Brain stem**	**D_1ccm_**	43.0	51.4	36.6	34.5	38.4	36.8	37.3	50
**Optic nerve le**	**D_1ccm_**	54.0	53.2	53.8	49.5	53.3	52.8	52.5	55
**Optic nerve ri**	**D_1ccm_**	53.1	50.4	53.0	49.1	52.1	51.5	51.8	55
**Chiasm**	**D_1ccm_**	29.5	30.2	27.2	27.5	25.5	24.9	25.5	50
**Bulbus oculi le**	**D_med_**	17.0	17.3	14.9	14.2	14.0	14.3	13.7	20
**Bulbus oculi ri**	**D_med_**	18.9	13.1	13.5	13.8	12.5	13.0	12.5	20
	**MU**	464.9	409.8	462.5	598.4	445.0	477.7	450.9	lower
	**treat. time (s)**	641	96	143	223	154	153	139	lower

D_max _is defined as dose to 1 ccm (D_1ccm_) of the structure volume. All dose values are given in Gy.

For the patients I, II and III, the clinical step-and-shoot IMRT plans were individually optimized using seven coplanar fields (0°, 51°, 103°, 154°, 206°, 257° and 308°) and for the patient IV nine coplanar fields of 6 MV were used (0°, 40°, 80°, 120°, 160°, 200°, 240°, 280°, 320°). For the optimization of single arc VMAT gantry angle spacing of 4° was selected and a maximal irradiation time of 150 seconds was allowed (s4°150s) with an arc ranging from 182° to 178°. The PTV was fully covered only for a collimator angle of 0° in all cases. For patient IV, s4°150 s started at 150° and stopped at 330° was performed with a collimator angle of 90 degrees and a couch angle of 270°, for dual arc VMAT an additional arc from 250° to 100° with collimator angle 0° and couch angle 0° was used. For the rest of the patients, one arc ranging from 178° to 182° was added for dual arc, allowing a time of 150 s per arc (d4°300s). For patient IV a non-coplanar 9-field IMRT plan (5 coplanar fields and 4 non-coplanar fields with couch angle 270°) was compared with the 9-field coplanar plan. Since there was no difference in PTV coverage between both plans and in terms of OAR sparing the plan with coplanar fields was similar or even slightly better than the non-coplanar plan, the coplanar plan was used for comparison.

To compare the impact of varying the time, following set up was performed: d4°200 s (dual arc with 4° gantry angle spacing, and 100 seconds per arc) and d4°400 s. To investigate the effect on the plan quality of modifying the gantry spacing, two additional plans were performed for each case: d2°300 s and d6°300 s.

### Plan evaluation

The evaluation of the plans was performed by means of the dose-volume-histograms and the dose distribution. For the PTV, D_95%_, D_5%, _the homogeneity H, defined as (D_5% _- D_95% _)/D_average _, and the conformity index (CI) (volume of the PTV receiving more than 95% of the prescribed dose divided by the total volume of the PTV) were evaluated. For the OARs spinal cord, brain stem, optic nerve and chiasm, D_1ccm _(dose to 1 ccm of the volume) was evaluated; for the parotid glands, D_median _is reported and for the bulbus oculi, D_mean _was evaluated.

### Dosimetrical verification

For dose measurement, the treatment plans were recalculated on a CT scan of the MatriXX Evolution^® ^(IBA Dosimetry, Schwarzenbruck, Germany) 2 D array, between slabs of RW3. It consists of 1020 vented pixel ionisation chambers in a square of 24.4 cm × 24.4 cm with a distance of 7.6 mm between chambers. As backscatter material RW3 was used. An investigation about the feasibility of hybrid plan verification has been published previously [26]. The planning system does not take the couch attenuation into consideration. To solve this inconveniency, the MatriXX Evolution^® ^has a gantry angle sensor, which allows to correct for angular dependencies including couch attenuation for each gantry position. Plan verification was performed for the twelve dVMAT plans with different gantry angle spacing of 2°, 4° and 6° (d2°300 s, d4°300 s, d6°300s). For patient IV it was not possible to perform the verification of the original plan because for the couch angle of 270°, the electronic system of the MatriXX would be irradiated. Therefore the plan was recalculated setting the couch angle to 0°. Evaluation of the verification was performed using the gamma criterion with 3% dose tolerance and 3 mm distance to agreement. The gamma criterion was considered fulfilled if γ < 1 in at least 95% of the pixels.

## Results

### Step-and-shoot IMRT, single arc and dual arc VMAT

**Figure 2:**
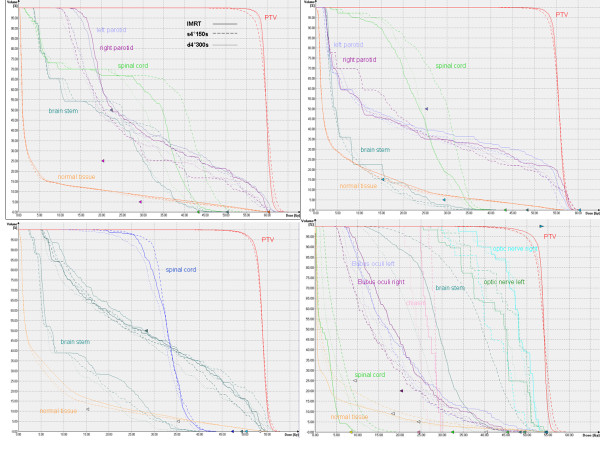
**Technique comparison study**. DVH comparison of IMRT, single arc VMAT and dual arc VMAT for all patients.

For this purpose, the IMRT plans were compared with the single arc VMAT plan and the dual arc plan (with gantry spacing 4° and maximal delivery time 300 s, which are the default values of the treatment planning system) in order to determine which technique can achieve a better plan quality. Figure 2 shows the DVHs for all patients and table 1 summarizes the results of PTV coverage and OAR doses for all patients.

Single arc VMAT failed to achieve the required target coverage and homogeneity in all cases, violating at the same time the tolerance dose for at least one OAR in all but one of the cases. Adding a second arc improved plan quality considerably, leading to similar results as IMRT. Target goal doses were achieved and OAR tolerance doses respected in all cases.

### Maximal delivery time

**Figure 3:**
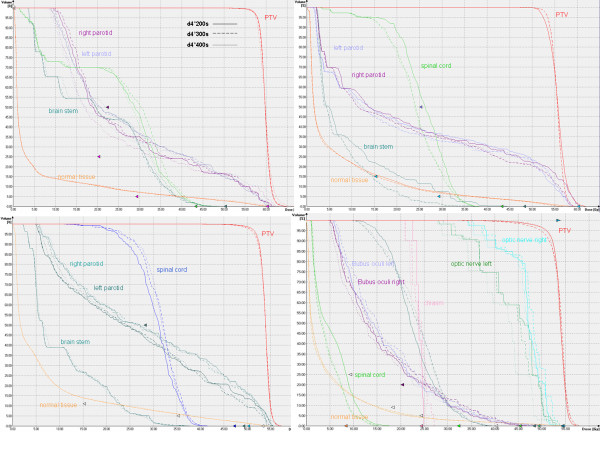
**Irradiation time comparison study**. DVH comparison of d4°200 s, d4°300 s and d4°400 s for patient all patients.

A comparison of d4°200 s, d4°300 s and d4°400 s was performed. Figure 3 shows the DVH for all patients. Table 1 shows the results of PTV coverage and OAR doses for all patients. No systematic influence of the delivery time on the plan quality was observed for the patient I and III. For patient II, reducing the time from 300 s to 200 s deteriorated the target coverage and OAR sparing, but the values were still below tolerance. For the last case, patient IV, the plan with 300 s showed a slightly inferior quality than the plans with 200 s and 400 s, but they were within the tolerances.

### Gantry angle spacing

**Figure 4:**
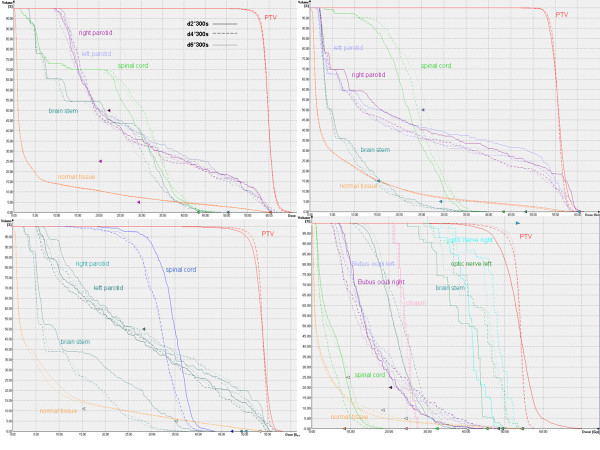
**Gantry angle spacing comparison study**. DVH comparison of d2°300 s, d4°300 s and d6°300 s for patient all patients.

The best plan quality was achieved with 4° and 6° gantry spacing, reducing the gantry spacing to 2° led to a deteriorated plan quality in some of the cases. Figure 4 shows the DVH comparison of the plans d2°300 s, d4°300 s and d6°300 s for all patients. Table 1 shows the results of PTV coverage and OAR doses for all patients. For patient I and II the modification of the number of control points achieved no plan quality improvement. Figure 4 shows that the DVHs were similar, but for the patient II, the dose to the OARs was slightly higher when applying 2°. For patient III, the plans with 4° and 6° achieved similar plan quality. When the gantry spacing was decreased to 2°, the DVH shows inferior target coverage and OARs dose sparing. The same effect was observed for patient IV; no acceptable target coverage could be achieved by using 2° gantry spacing, while the plans with 4° and 6° achieved a comparable dose distribution.

### Monitor units comparison and treatment time

The lowest number of MU per fraction dose was required for the single arc technique.

Dual arc resulted in similar or higher MU as IMRT, depending on the gantry spacing: The larger the gantry spacing, the lower the number of MU. Detailed information for each patient is given in table 1.

Regarding the treatment time it was observed that varying the gantry spacing does not affect the treatment time. The mean time for the plans d4°300 s was 198 s and 205 s for those with 6°. For the plans with 2°, the mean treatment time was 253 s. No significant difference was found between allowing the system 200, 300 or 400 seconds (from 300 s to 400 s), the irradiation time takes in average 227 s when allowing a maximal time of 200 s and 210 s when allowing a maximal time of 400 s. IMRT irradiation time was in average 702 s. Table 1 summarizes the irradiation time for each plan. Dual arc VMAT irradiation time is about one-third of IMRT time.

### Dosimetrical verification

**Figure 5:**
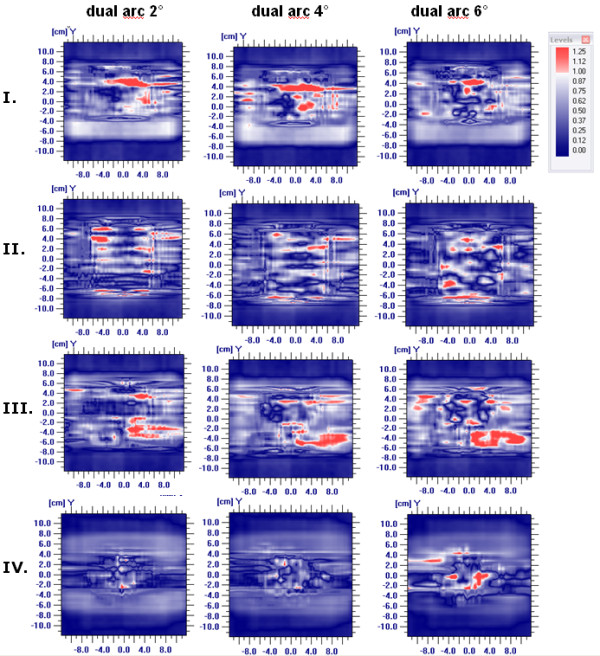
**Gamma evaluation**. Gamma evaluation of the gantry angle spacing comparison.

The results listed in table 2 show that the dosimetric verification of the plans with different gantry angle spacing showed good agreement of measured und calculated doses, passing the gamma test in all but one of the cases (patient III, with 4° and 6°). Averaged over all plans, the gamma evaluation was fulfilled in 97.2% of the pixels for the plans optimized with 2° gantry angle spacing, in 97.0% for the plans with 4° and 96.5% for the plans with 6°. Figure 5 shows the gamma evaluation for all patients.

**Table 2:** Gamma evaluation.

	d2°300s	d4°300s	d6°300s
**I**	96.60	95.35	98.06

**II**	96.56	97.95	97.09

**III**	95.83	94.92	92.80

**IV**	99.85	99.87	98.02

Gamma evaluation of the gantry spacing study with 3%, 3 mm criterion.

## Discussion

In our planning study we demonstrated that VMAT performed with a SynergyS^® ^linear accelerator is an appropriate technique to treat head and neck cancer. Recent planning studies have shown that VMAT could achieve conformal dose distributions for prostate and lung [10,11,27], which have a regular shape. For more complicated tumor sites as head and neck tumors, some studies revealed the requirement of an additional arc, because single arc does not succeed in achieving a plan quality comparable to IMRT [15,18,19]. On the other hand, other studies suggest that a single arc is good enough [6,21]. The results of the current investigation coincide with the mentioned VMAT studies for head and neck studies, which affirm that two arcs are required. In the present investigation it was first tried to fulfill the objectives planning with single arc, but no sufficient plan quality could be reached. Therefore, the study of the influence of the beam-on time and gantry angle spacing was performed with dual arc VMAT. It has been shown, that with the combination of Oncentra MasterPlan^® ^and Elekta linear accelerator, dual arc VMAT is required to achieve an acceptable plan quality.

The investigation of how the parameter maximal beam on time affects the plan quality showed that there is no identifiable difference in plan quality when increasing the treatment time. The plan quality became not better and the planning time increases. Furthermore, the additional time allowed for the plan d4°400 s was actually not used by the optimizer. Therefore, to use the default value of 150 seconds or even reducing it to 100 seconds per arc should be sufficient. Another factor which may affect the quality is the gantry angle spacing, which defines how many control points (or discrete segments) will be used for optimization and dose calculation. The continuous VMAT irradiation is approximated by discrete segments, the closer they are the better is the approximation to a continuous arc irradiation. Therefore, the agreement of measured and calculated dose is expected to increase when reducing the gantry angle spacing. Feygelman et al. [28] have confirmed this behavior when calculating the dose with a large spacing of 6° between control points. They found a dependence of the plan complexity on the sensitivity to the gantry angle spacing. Our investigation could not confirm this behavior. The results of our study reveal that the plan quality remains practically not affected when modifying this parameter (figure 4). For two patients, the plans with 2° achieved a lower plan quality. Particularly for the patient IV this effect was considerable. The reason can be the tip of the nose, because the algorithm has complications to calculate the dose. For 2° gantry angle spacing there are more segments in this region as for the plans with 4° and 6°, so there are more segments that can be affected by this effect. The dose validation shows that there is no dependence on the gantry spacing for the agreement of the dose calculation and the measured dose (table 2). Furthermore, reducing the gantry angle to 2° increases the calculation time by a factor of 1.6. For this reason, we conclude that planning with a gantry spacing of 4° or even 6° is reasonable. Some studies [15,18,19] have shown that VMAT reduces considerably the number of MU compared with IMRT. In our planning experience, the number of MUs of dual arc VMAT is similar as those of the IMRT. However, the MU of the step-and-shoot IMRT plans for the Elekta linear accelerator are lower than those presented in the mentioned studies for sliding window technique with a Varian machine [18,19].

We have presented four patient cases in which VMAT could have advantage to the patient compared with IMRT, dual arc VMAT with 4° gantry angle spacing and 300 seconds is a good compromise between plan quality, dose verification agreement and treatment time. Especially regarding the treatment time, table 1 shows that delivery time of IMRT takes about 12 minutes, for VMAT the time can be reduced by a factor of about 0.3 for dual arc and 0.15 for single arc. The quality assurance process is identical for IMRT and VMAT, but the IMRT plan delivery to the phantom takes longer than the VMAT delivery.

The treatment planning with the VMAT option becomes more complicated than for IMRT because of the high number of parameters which can be modified. The default values of VMAT planning for maximal treatment time per arc is 150 seconds and for the gantry angle spacing 4°. With this configuration, the treatment planning time increases by a factor of 7 when using single arc VMAT and a factor of about 14 for dual arc compared to IMRT.

It could be shown that dual arc with 4° gantry angle spacing and 150 seconds per arc is the best parameter set to achieve optimal combination of plan quality and treatment delivery time for head and neck cancer. However, these results are vendor-specific and similar comparison studies for other treatment planning systems and linear accelerators should be performed to generalize these results.

## Conclusions

Dual arc VMAT with Oncentra MasterPlan^® ^can achieve a comparable or superior plan quality to IMRT for all types of head and neck cancer included in this study. Both single arc and dual arc VMAT reduce the treatment time drastically compared with IMRT, but the plan quality of single arc was not sufficient. However, the cost of the improvement of the delivery time is that the calculation time increases. Allowing more treatment time does not actually improve the quality and increases the treatment planning time. The dosimetric validation has shown, that even performing the optimization with a large gantry angle spacing of 6° the results are as good as with 2° and 4°. Optimizing with larger gantry spacing could help to reduce the calculation time without compromising the plan quality.

## Competing interests

This work was partly supported by Theranostic, Solingen, Germany.

## Authors' contributions

JA carried out the comparison study, planned the IMRT and VMAT plans and drafted the manuscript. FP and OK helped to draft the manuscript and designed the medical aspects of it. BD participated in the design of the study and coordination of it and helped to draft the manuscript. All authors read and approved the final manuscript.
